# The complete chloroplast genome and phylogenetic analysis of *Syringa reticulata* subsp. *amurensis* (Rupr.) P.S.Green & M.C.Chang from Qinghai Province, China

**DOI:** 10.1080/23802359.2021.1934150

**Published:** 2021-06-03

**Authors:** Jiuli Wang, Tian Tian, Xia Han, Bin Ye, Xiuling Ma, Xianwen Meng, Jiuxiang Xie, Huakun Zhou

**Affiliations:** a College of Ecological Environment and Resources, Qinghai Nationalities University, Xining, China; b Qinghai Provincial Key Laboratory of Highvalue Utilization of Characteristic Economic Plants, Xining, China; c College of Agriculture and Animal Husbandry, Qinghai University, Xining, China; d Key Laboratory of Restoration Ecology in Cold Region of Qinghai Province, Northwest Institute of Plateau Biology, Chinese Academy of Sciences, Xining, China

**Keywords:** Evolutionary analysis, Oleaceae, plastome, *Syringa reticulata* subsp. *amurensis*

## Abstract

*Syringa reticulata* subsp. *amurensis* (Rupr.) P. S. Green & M. C. Chang (Oleaceae) is a shrub or tree with high medicinal value as well as great ecological significance as an urban garden plant. To better understand the molecular genetics and evolutionary of *S. reticulata* subsp. *amurensis*, its complete chloroplast genome was sequenced and annotated. The assembled chloroplast genome is a circular 156,141 bp sequence, consisting of 87,108 bp large single copy (LSC) region and 17,239 bp small single copy (SSC) region, which were flanked by a pair of 25,897 bp inverted repeats (IRs). The GC content of the chloroplast genome is 36.14%. Moreover, a total of 132 functional genes were annotated, including 88 protein-coding, 36 tRNA, and eight rRNA genes. Phylogenetic analysis showed that *S. reticulata* subsp. *amurensis* was most closely related to *S. reticulata* subsp. *Pekinensis* and the genus *Syringa* is paraphyletic group. This study provides important information for further phylogenetic studies on *S. reticulata* subsp. *amurensis* and its allies.

*Syringa reticulata* subsp. *amurensis* (Oleaceae: Syringeae), is a shrub or tree with high medicinal and horticultural values. Its flowers are white, luxuriant, and fragrant. *Syringa reticulata* subsp. *amurensis* can also be used to treat respiratory diseases (Zhu et al. 2021). In addition, with its excellent adaption to the strong environmental stress in northwest China, this species is considered a high-quality garden plant with great potential to improve urban ecology. Wild individuals of *S. reticulata* subsp. *amurensis* grow mainly in mixed forests on slopes and grasslands, or near gullies, 100–1200 m above sea level (Chang et al. 1996). The plant is usually cultivated as an ornamental in northern China (Chang et al. 1996). The chloroplast genome is particularly useful in studies on the maternal evolutionary history of angiosperms for its matrilinear inheritance without recombination (Nock et al. 2019). However, no studies on the complete chloroplast genome of *S. reticulata* subsp. *amurensis* have been published. In the present study, the complete chloroplast genome of *S. reticulata* subsp. *amurensis* was obtained using the next-generation sequencing (NGS) technologies and a phylogenetic analysis of *S. reticulata* subsp. *amurensis* and its allies was carried out.

The samples of *S. reticulata* subsp. *amurensis* were collected from Xining Botanical Garden, Xining City, Qinghai Province, China (36.62°N, 101.75°E). The experiment and data analysis followed Wang et al. (2019). The DNA was extracted from Silica gel dried young leaves (about 0.4 g) with a modified CTAB method (Doyle and Doyle 1987). The voucher specimen (specimen accession number: WangJL2019236, http://www.nwipb.cas.cn/znbm/qzgyswbbg/bbgjj/, Shilong Chen, herbarium@nwipb.cas.cn) was kept at the Qinghai-Tibetan Plateau Museum of Biology, Chinese Academy of Sciences (QTPMB). The genome sequencing was performed on an Illumina HiSeq Platform (Illumina, San Diego, CA) by Genepioneer Biotechnologies Inc. (Nanjing, China). Approximately, 5 GB of clean data were yielded. The sequencing reads were mapped to the reference chloroplast genomes using the Bowtie2 software (Langmead and Salzberg 2012). The SPAdes version 3.10.1 (Bankevich et al. 2012) and SSPACE version 2.0 (Boetzer et al. 2011) were used to assemble the chloroplast genome. The chloroplast genes were annotated with CpGAVAS (Liu et al. 2012) and the sequence coordinates for the genes were verified by BLAST search against the *Syringa reticulata* subsp. *pekinensis* (GenBank accession number: MN901632.1) reference chloroplast genome. Annotation errors were manually corrected.

The phylogenetic relationships of *S. reticulata* subsp. *amurensis* and its allies were inferred using the maximum-likelihood (ML) method based on the General Time Reversible (GTR) model (Nei and Kumar 2000). The sequences were aligned by MAFFT version 7.473 (Katoh and Standley 2013; online version: https://mafft.cbrc.jp/alignment/server/), and evolutionary analyses were performed in MEGA7 (Kumar et al. 2016). The evolutionary tree with the highest log likelihood (–464764.75) is shown (Figure 1). The bootstrap percentages of trees in which the associated taxa clustered together based on 1000 replicates are shown at the branch nodes. Initial trees for the heuristic search were obtained automatically by applying Neighbor-Joining and BioNJ algorithms to a matrix of pairwise distances estimated using the maximum composite likelihood (MCL) approach, and then topologies with superior log likelihood value were selected. The tree was drawn to scale, with branch lengths corresponding to the number of substitutions per site. The analysis involved 39 complete chloroplast genome sequences. There was a total of 202,491 positions in the final dataset.

**Figure 1.**
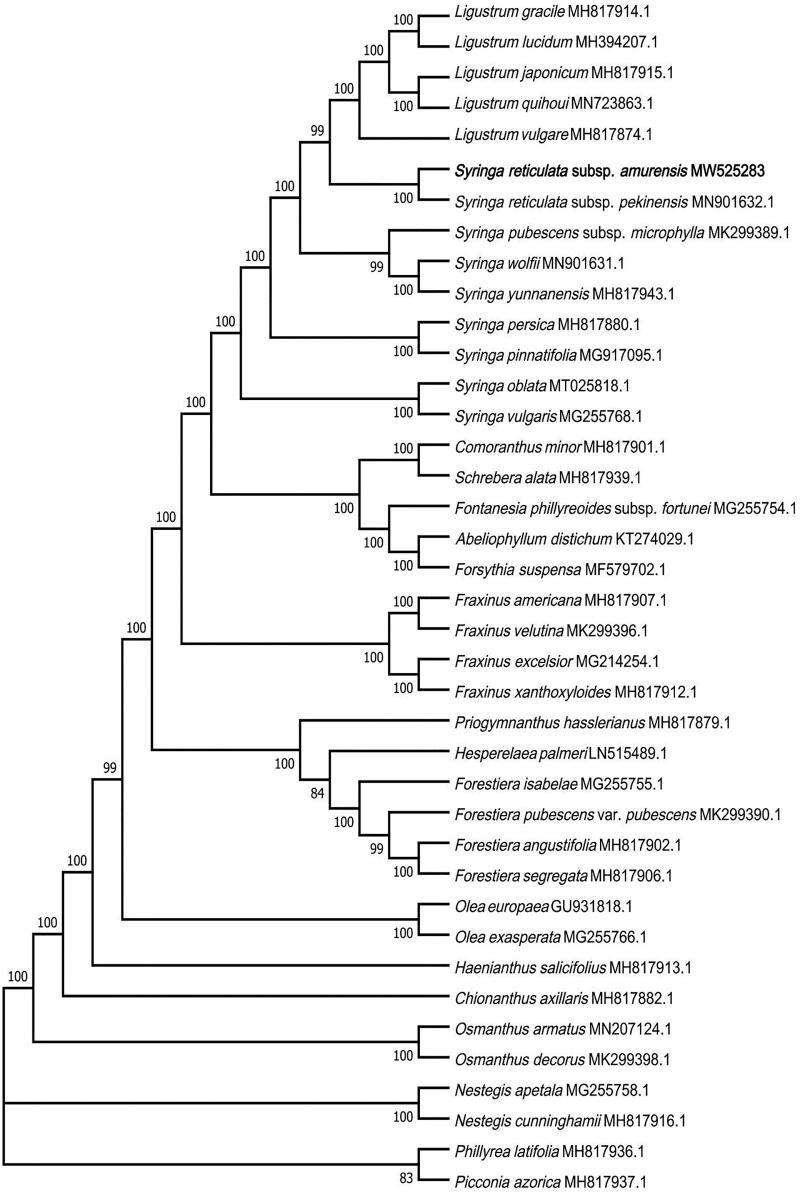
Maximum-likelihood (ML) tree of 39 species in the family Oleaceae based on the complete chloroplast sequences. Numbers above branches are bootstrap percentages (based on 1000 replicates).

The complete chloroplast genome of *S. reticulata* subsp. *amurensis* was 156,141 bp in length and has a typical quadripartite structure, containing a pair of IR regions of 25,897 bp, a large single copy (LSC) region of 87,108 bp, and a small single copy (SSC) region of 17,239 bp. The two IRs were separated by the LSC and the SSC. The GC content of the complete chloroplast genome was 38.03%. A total of 132 functional genes were annotated, including eight rRNA genes, 36 tRNA genes, and 88 protein-coding genes. The rRNA, tRNA, and protein-coding genes account for 6.06%, 27.27%, and 66.67% of all annotated genes, respectively.

The phylogenetic analysis fully resolved *S. reticulata* subsp. *amurensis* in a clade with *S. reticulata* subsp. *pekinensis* (Figure 1). The phylogenetic tree suggested that *S. reticulata* subsp. *amurensis* and *S. reticulata* subsp. *pekinensis* are polyphyletic with respect to *Ligustrum* spp. and other species classified to the genus *Syringa*. This result is consistent with previous studies (Li et al. 2002; Dupin et al. 2020; Wang et al. 2020). The present study provides important information for further molecular studies on *S. reticulata* subsp. *amurensis* and its allies.

## Data Availability

The genome sequence data obtained in this study are openly available in GenBank of NCBI at https://www.ncbi.nlm.nih.gov/ under the accession number MW525283. The associated BioProject, SRA, and Bio-Sample numbers are PRJNA692790, SRR13480493, and SAMN17717949, respectively.

## References

[CIT0001] Bankevich A, Nurk S, Antipov D, Gurevich AA, Dvorkin M, Kulikov AS, Lesin VM, Nikolenko SI, Pham S, Prjibelski AD, et al. 2012. SPAdes: a new genome assembly algorithm and its applications to single-cell sequencing. J Comput Biol. 19(5):455–477. 22506599 10.1089/cmb.2012.0021 PMC3342519

[CIT0002] Boetzer M, Henkel CV, Jansen HJ, Butler D, Pirovano W. 2011. Scaffolding pre-assembled contigs using SSPACE. Bioinformatics. 27:578–579. 21149342 10.1093/bioinformatics/btq683

[CIT0003] Chang MC, Qui LQ, Wei Z, Green PS. 1996. Oleaceae. In: Wu Z, Raven PH, editors. Flora of China. Beijing: Science Press and Missouri Botanical Garden; p. 272–319.

[CIT0004] Doyle JJ, Doyle JL. 1987. A rapid DNA isolation procedure from small quantities of fresh leaf tissues. Phytochem Bull. 19:11–15.

[CIT0005] Dupin J, Raimondeau P, Hong-Wa C, Manzi S, Gaudeul M, Besnard G. 2020. Resolving the phylogeny of the olive family (Oleaceae): confronting information from organellar and nuclear genomes. Genes. 11(12):1508. 10.3390/genes11121508 PMC7767060 33339232

[CIT0006] Katoh K, Standley DM. 2013. MAFFT multiple sequence alignment software version 7: improvements in performance and usability. Mol Biol Evol. 30(4):772–780. 23329690 10.1093/molbev/mst010 PMC3603318

[CIT0007] Kumar S, Stecher G, Tamura K. 2016. MEGA7: molecular evolutionary genetics analysis version 7.0 for bigger datasets. Mol Biol Evol. 33(7):1870–1874. 27004904 10.1093/molbev/msw054 PMC8210823

[CIT0008] Langmead B, Salzberg SL. 2012. Fast gapped-read alignment with Bowtie 2. Nat Methods. 9(4):357–359. 22388286 10.1038/nmeth.1923 PMC3322381

[CIT0009] Li J, Alexander JH, Zhang D. 2002. Paraphyletic *Syringa* (Oleaceae): evidence from sequences of nuclear ribosomal DNA ITS and ETS regions. Sys Bot. 27(3):592–597.

[CIT0010] Liu C, Shi L, Zhu Y, Chen H, Zhang J, Lin X, Guan X. 2012. CpGAVAS, an integrated web server for the annotation, visualization, analysis, and GenBank submission of completely sequenced chloroplast genome sequences. BMC Genomics. 13(1):715. 23256920 10.1186/1471-2164-13-715 PMC3543216

[CIT0011] Nei M, Kumar S. 2000. Molecular evolution and phylogenetics. New York (NY): Oxford University Press; p. 152–159.

[CIT0012] Nock CJ, Hardner CM, Montenegro JD, Termizi AAA, Hayashi S, Playford J, Edwards D, Batley J. 2019. Wild origins of macadamia domestication identified through intraspecific chloroplast genome sequencing. Front Plant Sci. 10:334. 30949191 10.3389/fpls.2019.00334 PMC6438079

[CIT0013] Wang J, Cao Q, Wang K, Xing R, Wang L, Zhou D. 2019. Characterization of the complete chloroplast genome of *Pterygocalyx volubilis* (Gentianaceae). Mitochondrial DNA Part B. 4(2):2579–2580. 33365634 10.1080/23802359.2019.1640644 PMC7706796

[CIT0014] Wang J, Wang W, Xiong P, Ye D, Jing M, Zhou H. 2020. The sequence and characterization of the complete plastome of *Syringa reticulata* subsp. *pekinensis* (Oleaceae). Mitochondrial DNA Part B. 5(2):2015–2017.

[CIT0015] Zhu W, Wang Z, Sun Y, Yang B, Wang Q, Kuang H. 2021. Traditional uses, phytochemistry and pharmacology of genus *Syringa*: a comprehensive review. J Ethnopharmacol. 226:113465. 10.1016/j.jep.2020.113465 33049343

